# 5-HTTLPR does not moderate the effect of attention bias modification for depression: a randomized sham-controlled trial

**DOI:** 10.1038/s41398-025-03581-3

**Published:** 2025-10-06

**Authors:** Ragnhild Bø, Rune Jonassen, Catherine J. Harmer, Øyvind Øverli, Hilde Loge Nilsen, Q. Ying Esbensen, Lisa Lirussi, Hallvard Heiberg, Brage Kraft, Eva Hilland, Tore C. Stiles, Vegard Øksendal Haaland, Nils Inge Landrø

**Affiliations:** 1 Clinical Neuroscience Research Group, Department of Psychology, University of Oslo, Oslo, Norway; 2 Department of Health Sciences, Oslo Metropolitan University, Oslo, Norway; 3 University of Oxford, PERL, Oxford, UK; 4 Department of Animal and Aquacultural Sciences, Norwegian University of Life Sciences (NMBU), Ås, Norway; 5 Department of Microbiology, Oslo University Hospital, Oslo, Norway; 6 Institute of Clinical Medicine, University of Oslo, Oslo, Norway; 7 Division of Medicine, Akershus University Hospital, Akershus, Norway; 8 Section for Physiology and Cell Biology, University of Oslo, Oslo, Norway; 9 Division of Psychiatry, Diakonhjemmet Hospital, Oslo, Norway; 10 Department of Behavioral Sciences, Oslo Metropolitan University, Oslo, Norway; 11 Department of Psychology, Norwegian University of Science and Technology, Trondheim, Norway; 12 Division of Mental Health, Sørlandet Hospital, Kristiansand, Norway

**Keywords:** Clinical genetics, Depression

## Abstract

The *5-HTTLPR* polymorphism in the serotonin transporter gene *SLC6A4* has previously been dubbed a plasticity marker. Within the *5-HTTLPR* polymorphism, a SNP (rs25531) in the L-allele in the promoter region, affects the transcription efficacy of the *SCL6A4*, leading to functionally important differences related to serotonin transporter availability in the synapses. *5-HTTLPR* has been implicated in magnitude of negative attentional bias, a causal risk factor for depression, and the modifiability of attentional biases both in positive and negative directions. Hence, this genotype may moderate the outcomes of attention bias modification (ABM) targeted at reducing depressive symptoms. We conducted a registered randomized sham-controlled trial of ABM in a sample of 301 participants with a history of Major Depressive Disorder (MDD) who had residual symptoms. They were randomized and underwent 14 days of two daily session of either ABM or sham at home. Of these, 264 provided genetic samples for determining the functionally important variant of the gene *SLC6A4*. We investigated if the SNP (*rs25531*) moderated the effect of ABM on symptoms of depression (HDRS, BDI-II) and anxiety (BAI), and attention bias post-intervention. None of the outcomes were moderated by the allelic variation in the promoter region of *5-HTTLPR*. Limitations include low level of depressive symptoms, lack of data on ethnicity, current and prenatal level of stress, and early traumatic experiences. The *5-HTTLPR* polymorphism did not moderate the effect of ABM on the symptom scales, nor attentional bias. Combination or interactions with other genes may be required for prescribing personalized interventions.

## Introduction

Attentional bias modification (ABM) is a digital intervention designed to modify negative attentional bias (AB [1]). AB is causally related to depressive symptoms [2] in all phases of Major depressive disorder (MDD), and modifying attentional bias through ABM has been shown to reduce residual symptoms of depression [3, 4].While trial outcomes are promising, the effect sizes tend to be small [5] or even non-existent [6], with the effect seemingly dependent on actual modification of attentional bias [7]. Some studies suggest that certain patient characteristics, such as levels of comorbid anxiety [8] and lower levels of inhibitory control [9], may modify the intervention efficacy. Another approach could be to explore whether genetic factors moderate the effects of ABM. The potential impact of genetic variations related to plasticity, affecting the individual’s sensitivity to both favorable and unfavorable environments [10] may be of relevance in the context of a targeted psychological intervention like ABM. The serotonin transporter polymorphism *5-HTTLPR*, is proposed to hold implications for personalized treatment [11], potentially supporting the broader goal of personalizing in depression interventions [12]. Therefore, ABM could serve as a potential intervention for reducing depressive symptoms by implicitly targeting the underlying attentional bias in specific subgroups of patients.

The human serotonin transporter is encoded by a single gene (*SLC6A4*). *5-HTTLPR* is a polymorphic sequence in the promotor region of *SLC6A4*. It is an indel, also containing a single-nucleotide polymorphism (SNP) in the insertion form only. From its regional location close to the promoter, the SNP (*rs25531*) influences the transcription of *SCL6A4* [13, 14]. r*s25531* affects the L allele and has two variants: L_A_ and L_G._ The low expressive short allele (S’: S and L_G_) is associated with low genetic expression and thereby less protein (fewer transporters), which implies higher concentrations of serotonin in the synaptic cleft. In contrast, the high expressive long allele (L; L_A_) implies higher concentrations of transporters, and thereby lower level of serotonin in the synaptic cleft. Based on being either heterozygote or homozygote carrier of these alleles, three levels of transcription efficiency can be differentiated, which are found to be functionally important for serotonin transmission in the brain [14]. While variants of the *5-HTTLPR* have been implicated in the magnitude of attentional bias [15], evidence also suggests that the magnitude of response to experimental manipulations is influenced by genetic makeup [16]. This metaanalysis suggests that experimental manipulations are more effective when participants have a susceptible genotype compared to a non-susceptible genotype, and they discuss that important main effects of interventions can be hidden by Gene x Environment (GxE) – interactions. In this context, ABM as a standardized and well-controlled intervention stands out as an ideal context for investigating GxE-interactions. Accordingly, this was studied by Fox and colleagues [10] who demonstrated that individuals without mental health disorders, having the low expressing, short variant of the *5-HTTLPR* polymorphism responded more profoundly to modification of attentional biases in both positive and negative directions compared to those with the high-expressing long variants. This finding indicates that *5-HTTLPR* should be considered a plasticity marker rather than related to susceptibility only, thereby holding implications for treatment outcomes.

In this preregistered study, we examined data from a randomized controlled trial that demonstrated an effect of ABM on clinician-rated depression symptoms [4]. We hypothesized that genetic variants would modify the effect of ABM compared to sham on four outcomes: reductions in-clinician rated and self-reported severity of depression, reductions in self-reported anxiety, and reductions in negative attentional bias - thought to be the mechanism underlying ABM.

## Materials & methods

This study is registered at Clinicaltrials.gov #NCT02658682.

### Participants

The parent trial included 301 participants fulfilling the criteria of Major Depressive Disorder (MDD) and having at least two prior episodes. Participants had primarily been treated as outpatients at a clinic in the Oslo-area. Individuals diagnosed with current- or former neurological disorders, psychosis, bipolar spectrum disorders, substance use disorders, attention deficit disorder, and head trauma were excluded. Details on the sample is provided in [4]. Of the 301, 264 were currently in remission and 37 had an ongoing depressive episode. Of the total sample, 263 consented to provide genetic data in the form of a swab sample to the Biobank “Genes in cognition and emotion” (REK Nord, t6/2006).

### Genetic analysis

To extract DNA from buccal epithelial cells, participants were instructed to gently rub an Isohelix SK-1S DNA Buccal Swab against the inner cheek for 1 min. The collected DNA samples were stored at room temperature until analysis. DNA isolation was conducted using either the DNeasy Blood and Tissue kit from Qiagen or the BFK-50 kit from Isohelix, according to their respective standard procedures.

Genotypes were reclassified into a functional model based on the 5-HTTLPR-directed level of transcriptional activity of *SLC6A4* as follows: L_G_/S, L_G_/L_G_ and S/S genotypes were classified as SS (low leveled RNA transcription); L_A_/S and L_A_/L_G_ genotypes were classified as LS (intermediate leveled); and L_A_/L_A_ genotype was classified as LL (high leveled).

### Intervention

All participants were randomized to receive either two weeks of ABM or a sham procedure. The specification of the intervention is detailed in [3, 4]. In short, through 28 5–7 min-sessions, twice daily for 14 days, they performed a 96-trials dot-probe task that included vertically presented pairs of facial stimuli (neutral, positive (happy) and negative (fearful and angry)) in equal numbers (negative-neutral, positive-neutral, and negative-positive). Sadness plays central role in depression; however, our ABM paradigm did not include sad stimuli. That said, angry and fearful faces also elicit amygdala activation, which is thought underlie attentional biases [17], thereby engaging neural circuits relevant for modification. After stimuli presentation (500 or 1 000 ms), participants identified the number of dots (∙or ∙∙ (the probe)) displayed in the same locations as one of the facial stimuli by pressing one of two designated keys on the keyboard as quickly and accurately as possible. In the ABM procedure, the probe was presented in the location of the relative more positive stimuli 87% of the time, whereas in the sham condition there were no contingency between type of stimuli and probe. The intervention was conducted at home on preprogrammed laptop computers provided by the research team.

### Attention bias (AB)

AB was assessed by one session of sham ABM the day before (pre) and the day after the intervention (post), including another set of stimuli than the one used during the interventional period. Hence, the participants in the sham condition underwent a maximum of 30 sessions, while the participants in the ABM condition were given two sessions of sham, one before and one after a maximum of 28 session of ABM. Pre and post AB was calculated based on the difference in reaction time to the probe in the location of the relatively more positive stimuli compared to reaction time to the probe when it was in the location of the relatively more negative stimuli [4].

### Randomization and blinding

Participants were told about the randomization procedure (ratio 1:1, based on a computerized random number generator) and that the trial aimed to explore the association between attention and mood yet remained unaware of their allocation and the distinctions between conditions. A separate laboratory technician programmed the laptops for intervention delivery and randomized participant based on a predetermined list. All evaluators remained unaware of participants’ treatment allocation, ensuring a double-blinded study design. Only after the completion of data collection was the randomization list disclosed.

### Measures

At baseline, to assess patients based on DSM-IV criteria, we used a semi-structured clinical interview, the MINI International Neuropsychiatric Interview PLUS 6.0.0 (M.I.N.I). The interviews were conducted by trained professionals or psychology students under supervision, all blind to study allocation. During the inclusion period, biweekly meetings to discuss cases and to uphold inter-rater reliability were performed.

We used Beck’s Depression Inventory-II (BDI-II [18]) to evaluate self-reported depression. Clinician-rated depression was assessed through the Hamilton Depression Rating Scale (HDRS [19]) by experienced clinical psychologists and psychology students trained on case exemplars. Bi-weekly supervision meetings were conducted to ensure consistent rating criteria and reach consensus if deviation occurred. Self-reported anxiety was measured using the Beck’s Anxiety Inventory (BAI; [20]). Participants self-reported on current use of SSRI/SNRI (0 = no, 1 = yes). Educational level is evaluated based on the International Standard Classification of Education (ISCED 2011).

### Statistical analysis

ANOVA and Chi-tests were used for investigating differences between demographic variables, symptom level, and use of medication between the genetic groups and for investigating the distribution of the genetic variant between the two conditions.

The Process macro [21] was used for investigating whether the functionally reclassified *5-HTTLPR* polymorphism (0 = SS’, 1 = LS’, 2 = LL’) moderated the effect of ABM (0 = sham, 1 = ABM) on the outcomes (change in HDRS, BDI-II, BAI and AB, operationalized as post-level – pre-level). The Process macro applies a linear regression model to the data, and we investigated if the relationship between the focal predictor (ABM) and the outcomes was dependent on the multicategorial moderator (dummy coded with SS as indicator). Both main effects and interaction effects were included in the model. Assumptions for the statistical tests were investigated. Leven’s test indicated homogeneity of variance between genotypes and residuals were normally distributed in all analysis. Significance level was set at *p* < 0.05. Tests were two-sided, and no corrections for multiple comparisons were made. Calculations conducted with G*Power 3.1.9.2, indicate that the current sample size could reveal medium sized moderator effects (f = 0.25), when Type-II error-probability was set to 0.15, with 5 predictor variables and 2 groups (ABM/sham).

### Ethical considerations

The study was approved by the Regional Committee for Health Research for South-Eastern Norway (REK Sør-Øst 2014/217), and all participants provided written informed consent. The biobank was approved by (REK Nord t6/2006). The authors assert that this work comply with the ethical standards of the relevant national and institutional committees on human experimentation and with the Helsinki Declaration of 1975, as revised in 2008.

## Results

### Participants

The sample consisted primarily of women with higher education (see Table 1). A minority were currently using SSRI/SNRIs, and most had subclinical levels of anxiety and depressive symptoms and limited negative attentional bias pre-treatment. There were no significant differences on variables of interest between the three genetic groups (see Table 2). In our sample, the genetic distribution was as follows: SS – 40 (13.3%); SL_G_ − 19 (6.3%); SL_A_ − 123 (40.9%); L_G_ L_G_ – 1 (0.3%); L_G_L_A_ - 25 (8.3%); L_A_L_A_ – 55 (18.3%). Hence, 42.2% of the participants had an S-allele, which is comparable to an expected ratio of S-alleles in a Caucasian sample (~40%). The genotype distribution was in Hardy-Weinberg equilibrium, χ^2^(1, *N* = 263) = 2.99, *p* > 0.05, Cramér’s *V* = 0.067.

**Table 1:** Demographic information dependent on genotype.

	SS (*n* = 59)	LS (*n* = 148)	LL (*n* = 56)	*p*	*V* / *η*^*2*^_*p*_
Sex (male/female)	14/45	45/103	18/38	*0*.55	0.032
Age	42.3 (13.8)	41.0 (13.0)	39.0 (13.0)	0.39	0.007
ISCED^a^	6.0 (0.9)	5.9 (1.2)	6.1 (1.1)	0.47	0.006
Current use of SSRI/SNRI (%)	11 (18.6)	44 (29.7)	16 (28.6)	0.26	0.031
HDRS pre	9.4 (5.6)	8.6 (5.3)	9.2 (6.2)	0.57	0.004
HDRS post	8.5 (6.1)	8.7 (5.6)	10.4 (11.2)	0.97	0.000
BDI-II pre	15.6 (11.5)	14.0 (9.5)	14.5 (10.7)	0.60	0.004
BDI-II post	12.2 (11.0)	11.3 (9.6)	10.4 (9.3)	0.65	0.003
BAI pre	9.9 (10.0)	9.0 (8.1)	10.1 (9.3)	0.64	0.003
BAI post	7.9 (8.3)	7.2 (7.2)	7.9 (8.7)	0.77	0.002
AB pre	−0.06 (27.7)	−0.02 (28.6)	−0.31 (25.9)	1.00	0.000
AB post	5.5 (21.1)	0.22 (16.6)	7.7 (14.9)	0.01	0.034

^a^ISCED level is missing for 13 participants.

*Note*. Values represent mean and standard deviation, or frequency. For categorical variables, *p*-values are based on Chi-square-tests, and for continuous variables univariate analysis of variance, with Cramér’s V and partial eta squared as corresponding effect size parameters, respectively.

*AB* attentional bias, *BAI* Beck’s anxiety inventory, *BDI-II* Beck’s depression inventory-II, *HDRS* hamilton depression rating scale, *SNRI* serotonin norepinephrine reuptake inhibitor, *SSRI* selective serotonin reuptake inhibitor.

**Table 2:** Distribution of genotype between the intervention allocations.

	Sham	ABM	All
SS’	27 (20.8%)	32 (24.1%)	59 (22.4%)
LS’	73 (56.2%)	75 (56.4%)	148 (56.3%)
LL’	30 (23.1%)	26 (19.5%)	56 (21.3%)
All	130 (49.4%)	133 (50.6%)	263 (100%)

*Note*. There was no significant difference between the distribution of genotype between the two conditions χ (2, *N* = 263) = 0.702, *p* = 0.70, Cramér’s *V* = 0.036.

### *5-HTTLPR* as moderator of ABM effect on depressive symptoms

#### HDRS - clinician rated depressive symptoms

**Fig. 1:**
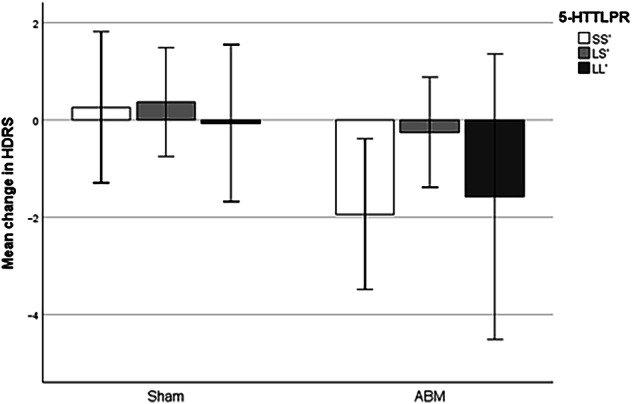
Effect of ABM on clinician-rated depressive symptoms dependent on 5-HTTLPR. *Note*. ABM Attentional bias modification, HDRS Hamilton Depression Rating Scale. There was no significant moderation effect from the 5-HTTLPR polymorphism. Error bars represent 95% CI.

With regards to change in HDRS as outcome variable, the overall model was non-significant [F (5, 257) = 1.401, *p* = 0.22, η^2^_p_ = 0.027]. ABM condition had a non-significant main effect on reductions in HDRS levels from pre- to post-intervention, *b* = −2.197, *t* = −1.703, *p* = 0.090, *CI* [−4.737, 0.344]. As SS’ was the indicator used for the analysis, these results represents the non-significant difference of having either ABM or sham if you have this genetic variant. LS’ had no main effect, *b* = 0.111 *t* = 0.100, *p* = 0.92, *CI* [−2.08, 2.30], Cohen’s d = 0.006, nor did LL’, *b* = −0.326, *t* = −0.249, *p* = 0.80, *CI* [−2.91, 2.25], Cohen’s d = 0.015. The overall interaction effect between *5-HTTLPR* and ABM on change in HDRS non-significant, *∆*R^2^ = 0.0044, F (2, 257) = 0.563, *p* = 0.56, η^2^_p_ = 0.004. There was not a significant interaction effect between ABM condition and LS’, *b* = 1.58, *t* = 1.032, *p* = 0.303, *CI* [−1.43, 4.58], Cohen’s d = 0.064, nor was there a significant interaction effect between ABM condition and LL’, *b* = 0.687, *t* = 0.372, *p* = 0.71, *CI* [−2.95, 4.33], Cohen’s d = 0.023. See Fig. 1 for bar chart visualizing the mean change in HDRS-levels dependent on ABM and 5-HTTLPR. Excluding participants currently using SSRI/SNRIs, did not affect the overall results.

#### BDI-II – self-reported depressive symptoms

**Fig. 2:**
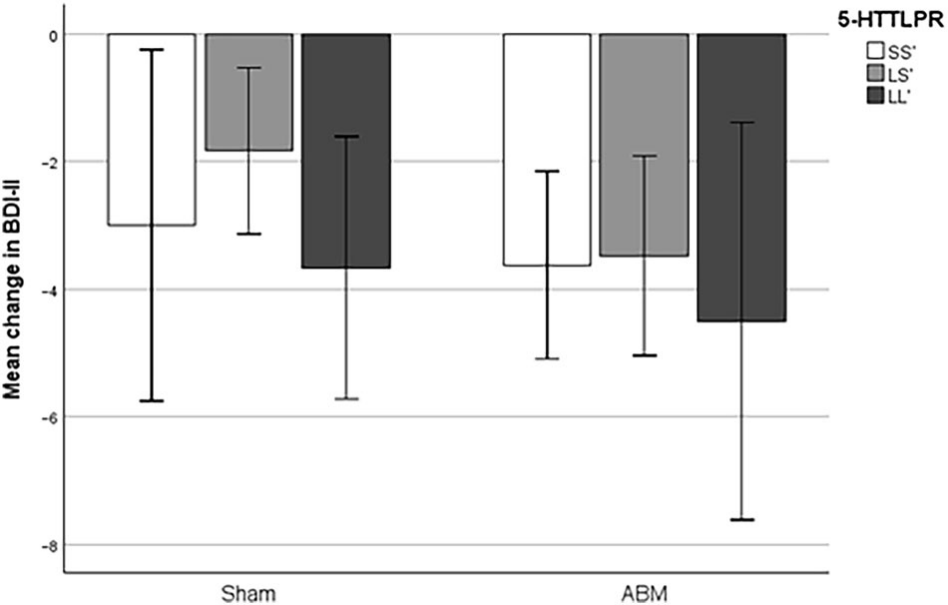
Effect of ABM on changes in self-reported depression symptoms dependent on 5-HTTLPR. *Note*. ABM Attentional bias modification, BDI-II Becks Depression Inventory-II. There was no significant moderation effect from the 5-HTTLPR polymorphism. Error bars represent 95% CI.

With regards to change in BDI-II as outcome variable, there were no statistically significant main effects or interaction effects to report. Therefore, the findings are presented in a condensed format only. None of the effects approached significance; all p’s > 0.41, all F’s < 1.1, and all t’s < +/- 0.8. Exclusion of participants currently undergoing psychopharmacological treatment, did not change the results substantially. See Fig. 2 for bar chart visualizing the mean change in BDI-II-levels dependent on ABM and *5-HTTLPR*.

### *5-HTTLPR* as moderator of ABM effect on anxiety symptoms

**Fig. 3:**
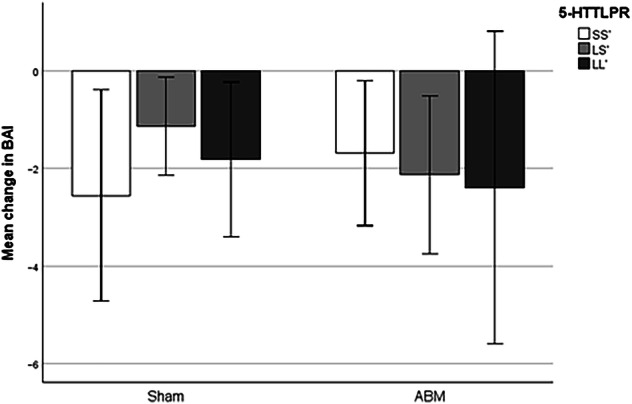
Effect of ABM on change in anxiety symptoms dependent on 5-HTTLPR. *Note*. ABM Attentional bias modification, BAI Becks Anxiety Inventory. There was no significant moderation effect from the 5-HTTLPR polymorphism. Error bars represent 95% CI.

Also, with regards to change in BAI as outcome variable, no moderating effect of *5-HTTLPR* was detected, and neither did any of the main effects approach significance; all p’s > 0.27, all F’s < 0.57, and all t’s < +/−1.2. Exclusion of participants currently undergoing psychopharmacological treatment, did not affect the results. See Fig. 3 for bar chart visualizing the mean change in BAI.

### *5-HTTLPR* as moderator of ABM effect on attentional bias

**Fig. 4:**
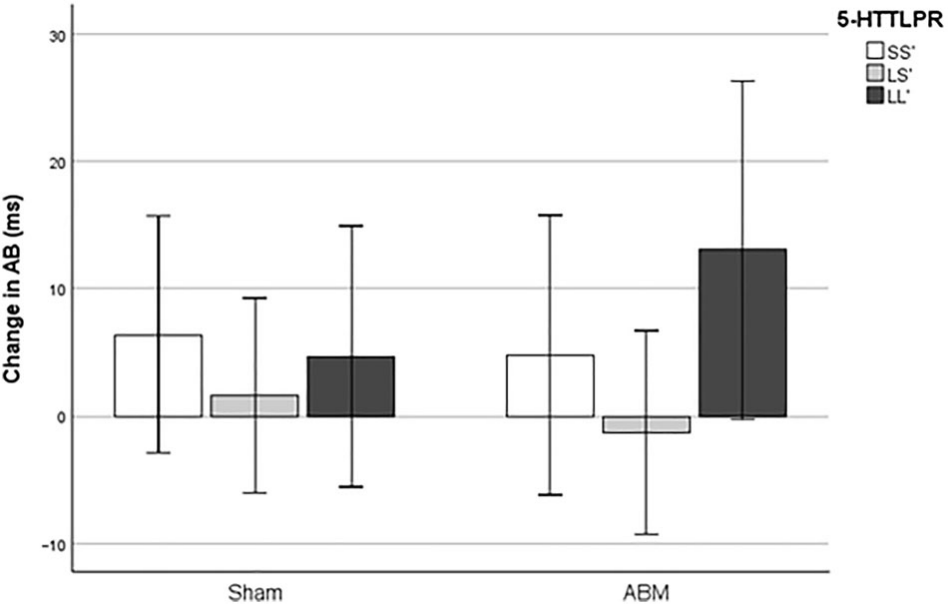
Effect of ABM on AB dependent on 5-HTTLPR. *Note*. AB attentional bias, ABM Attentional bias modification. Positive value means change away from negative material. There was no significant moderation effect from the 5-HTTLPR polymorphism. Error bars represent 95% CI.

Finally, with regards to change in negative AB as outcome variable, no significant results were found. All results were far from significant; all p’s >0.50, all t’s < +/−0.8, and all F’s < 0.82. When excluding participants currently using SSRI/SNRIs, the overall results were not affected. See Fig. 4 for a bar chart visualizing the mean change in attentional bias.

## Discussion

The main finding from this registered clinical trial suggests that the *5-HTTLPR* polymorphism did not moderate the effect of ABM compared to the sham procedure. This was observed when investigating change in self-reported or clinician-rated depressive symptoms, change in anxiety symptoms, and attentional bias away from negative material the proposed mechanisms underlying the procedure. These findings indicate that efficacy of the intervention is not influenced by genetic variation in the serotonin transporter polymorphism. Therefore, based on this study, *5-HTTLPR* does not appear to useful for personalizing ABM-treatment.

The field of therapygenetics, which explores the impact of genetic variations on psychological therapy outcomes, has provided some support for the use of single candidate genes (i.e., 5*-HTTLPR*) to predict treatment response [22]. However, growing evidence suggests that treatment outcomes – particularly in patients with treatment resistant depression and Major Depressive Disorder - may be better predicted by a combination [23] or an interaction [24] of multiple genetic polymorphisms rather than a single variant. Supporting this, studies have shown that a higher number of risk alleles moderate the negative effect of stress on proxies of depression (i.e., rumination [25]). Therefore, future studies should incorporate broader genetic datasets when investigating interventions to reduce residual symptoms in individuals with a history of MDD.

Our finding partly contradicts the findings in Fox, Zougkou [10] who reported that participants with the low expressive variant of *5-HTTLPR* were more sensitive to experimental manipulation of attentional bias than those with intermediate and high expressive participants. Their results suggested that individuals with the low expressive variant exhibit greater plasticity in response to *both* positive and negative environments. However, in our study, we found no association between *5-HTTLPR* and malleability of attentional biases towards more positive stimuli. Notably, we did not attempt to induce a negative attentional bias, which limits direct comparisons.

A key difference between the studies is the sample composition. First, our sample was more than twice as large, reducing the likelihood of extreme observations skewing the results. Second, our sample consisted of participants with a history of MDD compared to the sample without mental health disorders in Fox’s study. Since depression is associated with reduced plasticity [26], this may have constrained modification of bias. Consequently, the overall magnitude of bias change in our sample was smaller, and it was also in the opposite direction. Among the participants with the high-expressive genotype, the magnitude of change was the largest - though not statistically significant - compared to the other genetic variants.

We also did not find an association between *5-HTTLPR* and AB at baseline, which has previously been reported [15]. Several factors may explain this discrepancy. First, the reliability of the dot probe paradigm for assessing attentional bias has been repeatedly questioned [27], and in our overall sample, reliability estimates were low [28]. Since [15] also relied on the dot-probe paradigm, its poor reliability may have contributed to inconsistent findings. Second, differences in how studies classify genetic variants, particularly the inconsequent consideration of the L_G_L_A_ allelic variant, may have further contributed to the divergent results. Third, residual depressive symptoms in our sample might have affected reaction time estimates beyond the variation attributed to genetic factors. This suggest that future research should explore alternative methods for assessing AB.

Evidence suggests that recent exposure to stress and distal exposure to childhood trauma can affect vulnerability of carriers of the short allele of *5-HTTLPR* through increased methylation [29]. We lack data on exposure among our participants, and we therefore cannot exclude the possibility that they are unevenly distributed among the three genotypes and therefore influence our findings. Belsky and Gaspar have suggested that the plasticity conferred by the short variant of *5-HTTLPR* may be more profound during neuronal development than in adulthood [30]. Since we lack information on prenatal exposures, we cannot assess their potential role in shaping our results.

The generalizability of our findings to patients with more severe levels of depressive symptoms remains uncertain. While reduction of residual symptoms is important for reducing relapse risk [31], and presence of attentional bias in remitted phases of MDD may contribute to the relapse risk [3], the low levels of baseline symptoms also left minimal room for improvement. To enhance generalizability of our findings, future studies should also include patients with more severe symptoms.

We did not collect information on ethnicity in this study, however S-allele frequency in our sample indicates representativeness for a predominantly Caucasian population. Since previous studies have indicated that ethnicity may play a role in the relationship between genetic polymorphisms and treatment outcomes [32], the lack of such information is a limitation.

Moderation studies require a high number of participants to ensure adequate statistical power, and this study was powered to detect medium-sized moderation effects only. This indicates that smaller effect sizes would not reach statistical significance. At the same time, the effect sizes we did detect in the current study were indeed very small, raising doubts about their usefulness when personalizing treatment.

Summing up, the *5-HTTLPR* polymorphism did not moderate the effect of ABM when compared to a sham condition. The negative finding may indicate that there are other factors, like interactions or combinations of polymorphisms that may be more relevant for the effect of the intervention than a single genetic polymorphism.

## Data Availability

The data underlying this study are not publicly available due to privacy reasons, but is available from the corresponding author on reasonable request and with necessary ethical approval.
